# 经微导管碘化油化疗药乳剂栓塞治疗原发性富血型非小细胞肺癌41例分析

**DOI:** 10.3779/j.issn.1009-3419.2010.05.29

**Published:** 2010-05-20

**Authors:** 凌飞 罗, 洪武 王, 洪明 马, 珩 邹, 冬妹 李, 云芝 周

**Affiliations:** 100028 北京，北京煤炭总医院肿瘤微创治疗中心 Minimal Invasive Tumor Terapy Center, Meitan General Hospital, Beijing 100028, China

**Keywords:** 肺肿瘤, 碘化油, 栓塞化疗, 原发性, Lung neoplasms, Iodized oil, Chemoembolization, Primary

## Abstract

**背景与目的:**

动脉内化疗、栓塞是治疗肺癌的主要手段，但多数报道并未将小细胞肺癌与非小细胞肺癌、乏血型肺癌与富血型肺癌区分开来做进一步研究，栓塞前的化疗药灌注一直延用，对碘化油栓塞报道很少。本文通过对患者治疗后生存质量、临床有效率、生存期以及并发症进行总结，旨在探讨碘化油化疗药乳剂栓塞治疗原发性富血型非小细胞肺癌的临床疗效。

**方法:**

2008年1月-2009年1月经病理证实并完成随访的患者41例，CT增强扫描病灶中等度以上强化，提示血供丰富。包括中央型23例，周围型18例，鳞癌21例，腺癌15例，腺鳞癌5例；Ⅲb期34例，Ⅳ期7例。使用微导管在DSA下做靶动脉超选择插管，以液态碘化油+表阿霉素乳剂栓塞肿瘤毛细血管床，以明胶海绵微粒栓塞肿瘤供血动脉，液态碘化油用量5 mL-10 mL，表阿霉素10 mg-30 mg。最长随访12个月并与2007年-2009年相关文献进行对比。

**结果:**

治疗后患者症状改善，KPS显著提高（*P* < 0.05），局部病灶完全缓解（complete response, CR）0例，部分缓解（partial response, PR）31例，无变化（no change, NC）7例，疾病进展（progressive disease, PD）3例，总有效率（CR+PR）75.60%，临床受益率（CR+PR+NC）92.68%。总生存期超过12个月者33例（80.48%），Ⅲb期29/34（85.29%），Ⅳ期4/7（57.14%）。并发症脊髓损伤1例。

**结论:**

碘化油+化疗药乳剂单纯栓塞治疗原发性富血型非小细胞肺癌临床疗效肯定，同时避免化疗药毒副反应。DSA下使用微导管做绝对的超选择插管并仔细区分脊髓动脉以及肿瘤内动静脉瘘是避免脊髓损伤等严重并发症的关键。

## Abstract (en)

**Background and objective:**

Transcatheter arterial chemotherapy and embolization is the main method in the treatment of lung cancer, but most of the reports do not study individually to small cell lung cancer (SCLC), non-small cell lung cancer (NSCLC), hypovascular and hypervascular lung cancer. The pre-embolization perfusion of hemotherapeutics is still being used routinely and seldom report to iodized oil embolization. The article summarized the quality of life after the treatment, clinical efficiency, survival time and complications to evaluate the clinical effect of primary hypervascular NSCLC treated with embolization of emulsion of chemotherapeutics and iodized oil.

**Methods:**

The study totally analyzed 41 cases which confirmed by pathology and follow up study from January, 2008 to January 2009. The CT scan with Ⅳ contrast demonstrates over moderate enhanced lesion which indicate hypervascular. Within the 41 cases, 23 cases of central, 18 cases of peripheral. Suqamous carcinoma 21 cases, adenocarcinoma 15 cases and squamoadenocarcinoma 5 cases. Stage Ⅲb 34 cases, stage Ⅳ 7 cases. Superselective incubation with microcatheter under DSA, to embolize the capillary bed with liquefied iodized oil and the emulsion of pharmorubicin, to embolize the supply artery of the tumor with gelatin foam microparticle. The liquefied iodized oil 5 mL-10 mL, pharmorubicin 10 mg-30 mg. The longest follow up is 12 months and to compare with the references of 2007-2009.

**Results:**

The KPS is widely acclaimed after the treatment (*P* < 0.05), no complete response (CR), 31 cases of partial response (PR), 7 cases of no change (NC) and 3 cases of progressive disease (PD), the total efficiency (CR+PR) is 75.6%. The clinical efficiency (CR+PR+NC) is 92.68%. 33 cases of total survival tome over 12 months (80.48%), Ⅲb stage 29/34 (85.29%), Ⅳ stage 4/7 (57.14%). 1 case with severe complication-spinal injury.

**Conclusion:**

To treat primary hypervascular NSCLC with simple embolization of emulsion of chemotherapeutics and iodized oil is very useful and can avoid the side effect of chemotherapeutics. The key point to avoid spinal injury and other severe complications is to distinguish the spinal aretery and intratumor AV fistula by using superselective incubation with microcatheter under DSA.

20世纪70年代动脉内灌注化疗就已应用于肺癌的治疗并取得一定的近期临床疗效，截至今日动脉内化疗、栓塞已成为肺癌治疗的重要手段之一^[1]^。本文旨在探讨经微导管碘化油+化疗药乳剂栓塞治疗原发性富血型非小细胞肺癌的临床疗效。

## 1 材料与方法

### 1.1 主要资料

2008年1月-2009年1月共治疗48例病理证实的富血型原发性非小细胞肺癌，失访7例。完成随访的41例中，男性29例，女性12例，平均年龄62.1岁±1.4岁。中央型23例，周围型18例。鳞癌21例，腺癌15例，腺鳞癌5例。Ⅲb期34例，Ⅳ期7例。48例患者KPS为53.542±8.627，38例伴有咯血或痰中带血。

### 1.2 适应证及操作方法

#### 1.2.1 适应证

全部48例患者无血管内介入治疗禁忌症，治疗前均经CT增强扫描提示血供丰富，肿瘤强化后CT值较平扫最低增加30。

#### 1.2.2 靶动脉栓塞化疗

DSA下Seldinger技术穿刺股动脉，5fCobra（C3）或西盟导管行靶动脉超选择插管，DSA明确肿瘤供血动脉及肿瘤染色后，使用微导管做进一步超选择插管直达肿瘤。肿瘤血供主要来源于支气管动脉、肋间动脉、胸廓内动脉及甲状颈干分支等体动脉。支气管动脉插管成功后首先经造影导管推注地塞米松5 mg。微导管位置准确后间断推注碘化油+化疗药乳剂栓塞肿瘤毛细血管床，再以明胶海绵微粒栓塞肿瘤供血动脉主干。液态碘化油用量5 mL-10 mL，表阿霉素10 mg-30 mg。

#### 1.2.3 随访

最长随访12个月，对患者治疗后生存质量、临床有效率、生存期以及并发症进行总结。

## 2 结果

48例操作技术成功率100%。治疗后第1天38例咯血或痰中带血患者21例出血量减少，17例出血停止。治疗后2周评价并CT复查，KPS为77.292±11.249，较治疗前提高，差异具有统计学意义（*t*=-17.564, *P* < 0.001），肿瘤内均可见高密度碘化油浓聚（图 1）。

**1:**
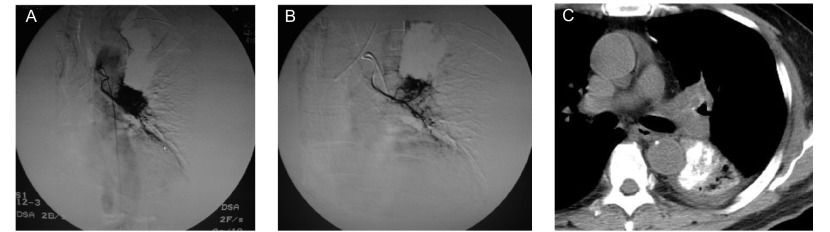
左下叶腺癌单纯碘化油乳剂栓塞化疗。A：左侧支气管动脉DSA显示肿瘤浓染；B：微导管超选择插管后栓塞化疗；C：治疗后CT显示肿瘤内碘化油浓聚 Adenocarcinoma of left inferior lobe with simple embolization of emulsion of chemotherapeutics and iodized oil. A: Left bronchial artery DSA demonstates conspicuous tumor stain; B: Embilization and chemotherapy after superselective incubation with microcatheter; C: After the treatment, CT demonstrates iodized oil collection in tumor

48例栓塞前DSA均未见明确发卡样脊髓动脉显示，6例右侧中央型肺癌栓塞后DSA见肋间动脉干大量紊乱血管支充盈（图 2）。并发症脊髓损伤1例（2.08%），为右上叶中央型肺癌（图 3），经积极处理，2周后部分恢复，大、小便可控制，下肢肌力升至Ⅲ级和Ⅲ级加。

**2:**
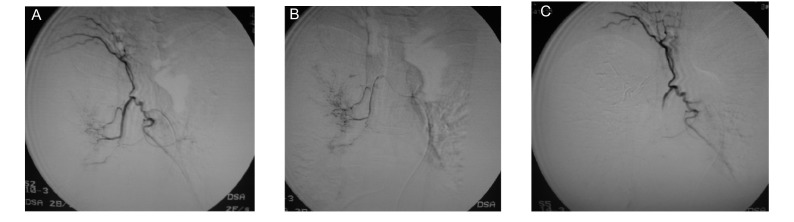
右上叶鳞癌单纯碘化油乳剂栓塞化疗。A：右侧支气管动脉与肋间动脉共干，支气管动脉远端见肿瘤血管及染色；B：微导管超选择插管后栓塞化疗；C：肿瘤血管栓堵后肋间动脉干大量紊乱纤细血管支充盈 Squamocarcinoma of left superior lobe with simple embolization of emulsion of chemotherapeutics and iodized oil. A: Right bronchial artery and intercostals artery share one trunk, the distal area of bronchial aretery demonstrates tumor artery and tumor stain; B: Embilization and chemotherapy after superselective incubation with microcatheter; C: After the embolization, the trunk of intercostal artery demonstrates plenty of disorganized branches filling

**3:**
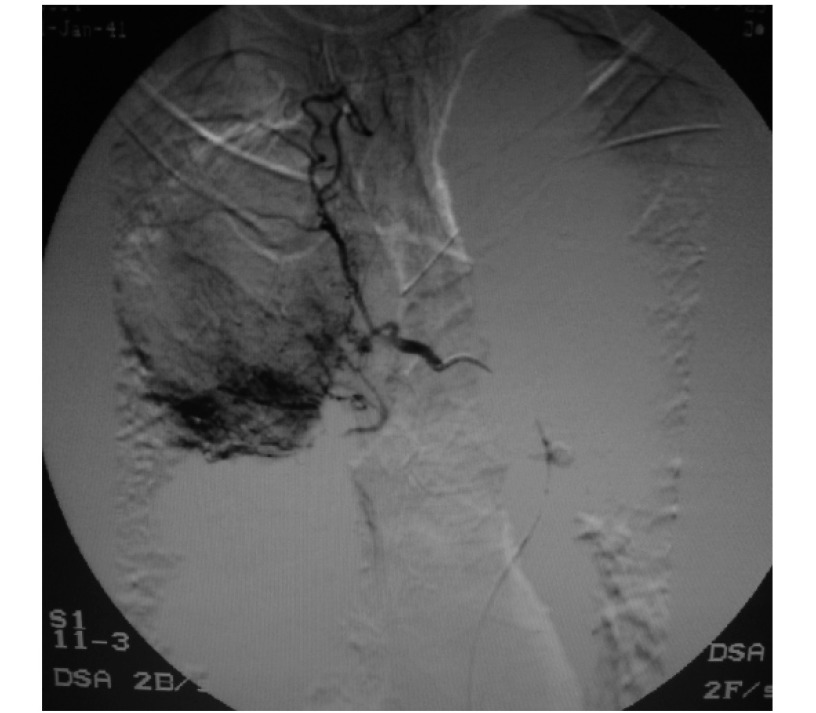
右上叶中央型鳞癌。右侧支气管动脉与肋间动脉共干，肋间动脉干同时见扭曲血管支充盈 Central squamocarcinoma of right superior lobe. Right bronchial artery and intercostals artery share one trunk, some tortuous branches of the trunk of intercostals artery filling

41例完成12个月随访，局部病灶完全缓解（complete response, CR）0例，部分缓解（partial response, PR）31例）（图 4），无变化（no change, NC）7例，疾病进展（progressive disease, PD）3例，总有效率（CR+PR）75.60%，临床受益率（CR+PR+NC）92.68%。总生存期超过12个月者33例（80.48%），Ⅲb期29/34（85.29%），Ⅳ期4/7（57.14%）。

**4:**
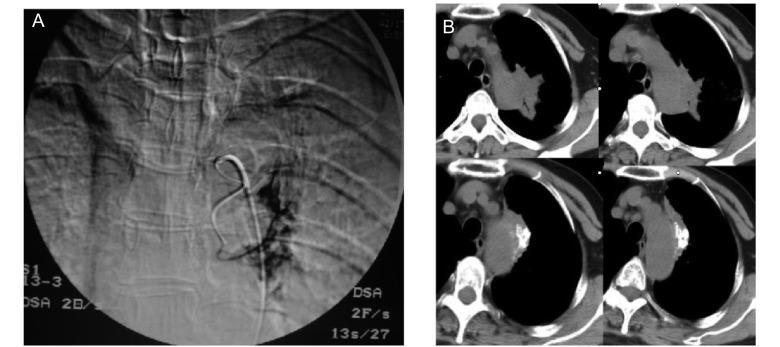
左上叶腺癌单纯碘化油乳剂栓塞化疗。A：微导管超选择插管后栓塞化疗；B（上）：肿瘤治疗前；B（下）：治疗后4个月复查肿瘤内碘化油浓聚，瘤体缩小 Adenocarcinoma of left superior lobe with simple embolization of emulsion of chemotherapeutics and iodized oil. A: Embilization and chemotherapy by superselective incubation with microcatheter; B (top): pre-treatment; B (bottom): Follow up 4 months after treatment, CT demonstrates iodized oil collection in tumor and the tumor become smaller

## 3 讨论

原发性非小细胞肺癌对全身放、化疗不敏感，长期随访结果显示动脉内大剂量灌注化疗与全身化疗相比，二者远期临床疗效并无显著差异。截至目前动脉内介入放射学研究的多数报道也并未对小细胞肺癌和非小细胞肺癌、乏血型肺癌和富血型肺癌做进一步的分类研究。

首过效应和局部高浓度是灌注化疗的理论依据^[2]^，但对于非小细胞肺癌，化疗药的毒副作用更应该值得关注。锲利宇等总结200例肺癌灌注化疗，并发症包括消化道反应（86.5%）、骨髓抑制（16.0%）、肾脏毒性（7.5%）、心脏毒性（6.5%）、肝脏毒性（3.0%）、周围神经毒性（0.5%）、脊髓损伤（0.5%）。杨熙章等^[3]^研究认为高浓度的毒性药物可能是导致脊髓损伤的主要因素。本组治疗放弃了大剂量的化疗药灌注，仅以少量ADM混合液态碘化油实施肿瘤毛细血管床栓塞，从根本上避免了化疗药的毒副作用。

碘化油+化疗药乳剂栓塞已是治疗原发性肝癌的首选，肺癌的栓塞效果也与栓塞部位密切相关。颗粒型栓塞剂仅能做主干栓塞或小动脉栓塞，液态碘化油则可栓塞肿瘤毛细血管床即达到终末栓塞，使肿瘤侧枝循环难以建立^[4]^。此外，碘化油携带化疗药较长时间滞留于肿瘤内，化疗药缓慢释放，则保证了肿瘤内较长时间的化疗药高浓度作用，使肿瘤缺血坏死更为彻底。碘化油栓塞的并发症除脊髓损伤外，当肿瘤内存在动静脉瘘时，碘化油可直接回流左心进入体循环而引起严重的异位栓塞^[5]^。相关的研究结果显示，DSA、手推造影、治疗前使用地塞米松以及利多卡因脊髓功能诱发试验等均是防止发生脊髓损伤的有效方法，除此之外，我们的体会是微导管的使用同样不可忽视。对于富血肿瘤，由于血流动力学因素，普通造影导管造影时大部分造影剂随血流充盈肿瘤血管，此时相对纤细的脊髓支往往不能显示。所以，不管普通造影导管DSA是否有脊髓支显示，均应以微导管做绝对的超选择插管直达肿瘤。普通造影导管插管不宜过深，能支撑微导管做超选择插管即可，加之微导管纤细、柔软，二者结合即可防止发生血管痉挛使治疗不能继续，还可最大限度保护血管内膜，为下一次治疗创造有利条件。对于右肺上叶中央型肺癌，我们的体会是应谨慎使用碘化油乳剂栓塞，因为右支气管动脉、肋间动脉和脊髓前动脉往往共干，即便微导管准确到位，也多由于插管深度不够而使碘化油乳剂极易返流。

本组病例治疗后临床症状缓解，生存质量提高。总有效率（CR+PR）为75.60%，临床受益率为（CR+PR+NC）92.68%。12个月生存33例（80.48%）。检索2007年-2009年CHKD期刊关于肺癌栓塞治疗文献11篇，总有效率为50%-69.7%，12个月生存率为78.7%。本组总有效率、12个月生存率均略高于所检索文献，但二者对比并无统计学差异，我们考虑原因主要在于本组病例不包括小细胞肺癌，小细胞肺癌对全身放/化疗敏感，局部治疗效果更优。

综上所述，我们认为经微导管碘化油+化疗药乳剂栓塞治疗原发性富血型非小细胞肺癌临床疗效肯定，同时可避免化疗药毒、副反应。DSA下使用微导管做绝对的超选择插管并仔细区分脊髓动脉以及肿瘤内动-静脉瘘是避免脊髓损伤和异位误栓等严重并发症的关键。
